# An Antibody Screen of a *Plasmodium vivax* Antigen Library Identifies Novel Merozoite Proteins Associated with Clinical Protection

**DOI:** 10.1371/journal.pntd.0004639

**Published:** 2016-05-16

**Authors:** Camila T. França, Jessica B. Hostetler, Sumana Sharma, Michael T. White, Enmoore Lin, Benson Kiniboro, Andreea Waltmann, Andrew W. Darcy, Connie S. N. Li Wai Suen, Peter Siba, Christopher L. King, Julian C. Rayner, Rick M. Fairhurst, Ivo Mueller

**Affiliations:** 1 Population Health and Immunity Division, Walter and Eliza Hall Institute of Medical Research, Melbourne, Australia; 2 Department of Medical Biology, University of Melbourne, Melbourne, Australia; 3 Laboratory of Malaria and Vector Research, National Institute of Allergy and Infectious Diseases, National Institutes of Health, Bethesda, Maryland, United States of America; 4 Malaria Programme, Wellcome Trust Sanger Institute, Hinxton, United Kingdom; 5 Center for Outbreak Analysis and Modelling, Department of Infectious Disease Epidemiology, Imperial College London, London, United Kingdom; 6 Vector Borne Diseases Unit, PNG Institute of Medical Research, Madang, Papua New Guinea; 7 National Health Training & Research Institute, Ministry of Health, Honiara, Solomon Islands; 8 Center for Global Health & Diseases, Case Western Reserve University, Cleveland, Ohio, United States of America; 9 ISGlobal, Barcelona Ctr. Int. Health Res. (CRESIB), Hospital Clínic, Universitat de Barcelona, Barcelona, Spain; Queensland Institute of Medical Research, AUSTRALIA

## Abstract

**Background:**

Elimination of *Plasmodium vivax* malaria would be greatly facilitated by the development of an effective vaccine. A comprehensive and systematic characterization of antibodies to *P*. *vivax* antigens in exposed populations is useful in guiding rational vaccine design.

**Methodology/Principal Findings:**

In this study, we investigated antibodies to a large library of *P*. *vivax* entire ectodomain merozoite proteins in 2 Asia-Pacific populations, analysing the relationship of antibody levels with markers of current and cumulative malaria exposure, and socioeconomic and clinical indicators. 29 antigenic targets of natural immunity were identified. Of these, 12 highly-immunogenic proteins were strongly associated with age and thus cumulative lifetime exposure in Solomon Islanders (*P*<0.001–0.027). A subset of 6 proteins, selected on the basis of immunogenicity and expression levels, were used to examine antibody levels in plasma samples from a population of young Papua New Guinean children with well-characterized individual differences in exposure. This analysis identified a strong association between reduced risk of clinical disease and antibody levels to P12, P41, and a novel hypothetical protein that has not previously been studied, PVX_081550 (IRR 0.46–0.74; *P*<0.001–0.041).

**Conclusion/Significance:**

These data emphasize the benefits of an unbiased screening approach in identifying novel vaccine candidate antigens. Functional studies are now required to establish whether PVX_081550 is a key component of the naturally-acquired protective immune response, a biomarker of immune status, or both.

## Author Summary

*Plasmodium vivax* is now the predominant malaria parasite outside Africa. Because *P*. *vivax* can remain dormant in the liver for months, identifying and treating *P*. *vivax* in asymptomatic individuals is difficult. Additionally, current widely-used vector control measures are less efficient against mosquitoes that transmit *P*. *vivax*. An effective vaccine would therefore immensely facilitate *P*. *vivax* elimination. Unfortunately, little is known about *P*. vivax biology and only a few proteins have been investigated as targets for vaccine development. To address these knowledge gaps, we measured antibody levels to 34 entire ectodomain proteins predicted to be involved in *P*. *vivax* invasion of erythrocytes, in samples from individuals living in 2 malaria-endemic Asia-Pacific countries. We found that antibodies in malaria-exposed Solomon Islanders were reactive to the majority of proteins in our panel, and that antibodies to 12 of these proteins strongly reflected cumulative life-time exposure to *P*. *vivax*. In samples from Papua New Guinea children, we identified an association between antibodies to 3 proteins and protection against clinical malaria. Our results demonstrate that screening antibodies to a large number of *P*. *vivax* proteins is a useful approach in identifying novel targets of immunity. Functional studies are now required to establish whether these proteins are biomarkers of an individual’s immune status, potential vaccine candidates that warrant further development, or both.

## Introduction

Intensified research and funding have helped to significantly reduce the morbidity and mortality of malaria, and an increasing number of countries are now aiming to eliminate this disease [1–3]. In Asia-Pacific and the Americas, however, interrupting local *Plasmodium vivax* transmission will be particularly challenging. The ability of *P*. *vivax* to form dormant liver hypnozoites, which are responsible for ~80% of all blood-stage infections [4, 5], provides a source of new blood-stage infections in the absence of transmission. *P*. *vivax* commonly causes low-density asymptomatic infections that often go undetected and thus untreated. Moreover, the early maturation and peripheral circulation of *P*. *vivax* gametocytes, coupled with high infectivity and rapid development in mosquitoes, make *P*. *vivax* more refractory to control measures [6]. As a consequence, *P*. *vivax* is now the predominant *Plasmodium* species outside Africa [1].

New tools are needed to control and eliminate *vivax* malaria. Vector control strategies that are broadly effective in reducing *P*. *falciparum* transmission, such as insecticide-treated nets (ITNs) and indoor residual spraying, seem to be less effective against *P*. *vivax* vectors [7, 8], which are more likely to bite and rest outdoors, and less likely to bite humans than African *P*. *falciparum* vectors [9]. Furthermore, primaquine, the only drug effective against *P*. *vivax* hypnozoites, is associated with hemolysis in glucose-6-phosphate dehydrogenase-deficient individuals [10]. Similar effects have been seen for tafenoquine, the only other liver-stage drug in clinical development [11]. Given these challenges, the development of a highly effective vaccine would immensely facilitate *P*. *vivax* elimination, perhaps even more so than *P*. *falciparum* elimination [12].

Merozoites represent the only extracellular phase of the *Plasmodium* blood-stage life cycle, and merozoite antigens are therefore appropriate vaccine targets. Several studies have investigated merozoite antigens as targets of natural protective immunity to *P*. *falciparum* malaria [13], and their potential as vaccine candidates [14]. For *P*. *vivax*, the availability of the genome sequence [15] and transcriptome [16] have enhanced our understanding of this parasite’s biology, facilitating the identification of many proteins that are homologous to *P*. *falciparum* antigens [17–19]. However, the targets of natural immunity to *P*. *vivax* malaria remain poorly understood, and systematic screens of multiple antigens are lacking [20]. As a consequence, there are currently only a handful of *P*. *vivax* vaccine candidate antigens in pre-clinical development, with only a single blood-stage antigen (PvDBP) nearing clinical development [21].

In this study, we investigated 34 recombinant *P*. *vivax* protein ectodomains [22], known or predicted to localize to the merozoite cell surface, micronemes, or rhoptries, as targets of natural immunity. For 12 highly-immunogenic proteins, we investigated associations between levels of antibodies and indicators of current and cumulative malaria exposure in a moderately-endemic area of the Solomon Islands (SI). Using a cohort of young Papua New Guinean (PNG) children with well-characterized individual differences in exposure, we identified an association between reduced incidence of clinical disease and antibody levels to 3 proteins, including a novel hypothetical protein that has not been previously studied. These data emphasize the benefits of an unbiased screening approach in identifying vaccine candidates and indicate that these 3 antigens are high-priority targets for further functional studies, and potentially vaccine development.

## Methods

### Protein library

Proteins were designed, constructed, and expressed as described previously for *P*. *falciparum* merozoite proteins [23, 24]; the *P*. *vivax* ectodomain library has been described in detail by Hostetler et al. (S1 Table) [22]. Briefly, sequences derived from the *P*. *vivax* Salvador-1 strain encoding merozoite ectodomains, excluding their signal peptide, transmembrane domain, and glycosylphosphatidylinositol (GPI) anchor sequences (if present), were codon-optimized for expression in human cells and chemically synthesized (GeneArt AG). Soluble recombinant proteins (S1 Table) containing a ~25-kDa C-terminal rat Cd4d3+d4 (Cd4) tag were expressed in human embryonic kidney (HEK) 293E cells as either biotinylated or 6-His-tagged forms, culture supernatants were collected 6 days after transfection, and biotinylated proteins were dialysed in HEPES-buffered saline. All expression plasmids are openly available at Addgene (http://www.addgene.org/express/vivax/).

### Protein purification

6-His-tagged proteins were purified by immobilized metal-ion affinity chromatography using HisTrap-HP columns on an AKTA Xpress (GE Healthcare) following the manufacturer’s instructions. Proteins were then conjugated to Luminex Microplex microspheres (Luminex Corporation) as described [25], using the following concentrations per 2.5x10^6^ beads: P41, 0.5 μg/mL; PVX_081550, 1.2 μg/mL; P12, 0.2 μg/mL; GAMA, 0.015 μg/mL; ARP, 0.09 μg/mL; CyRPA, 1.5 μg/mL; and Cd4, 2 μg/mL. Coupling efficiency was determined by using an immune plasma pool known to be highly reactive with the antigens, with the appropriate antigen concentration resulting in high fluorescence intensity by the reporter fluorochrome.

### Study populations

#### Immunoreactivity and comprehensive screens

Samples collected in a cross-sectional survey (3501 individuals aged ≥6 months) in May 2012 in Ngella, Central Island Province, SI were used [26]. A random subset of 22 adolescents (10–19 years) and 24 adults (20–50 years) were selected for the immunoreactivity screen, because they had a higher cumulative exposure and were thus more likely to have acquired substantial levels of natural immunity. In all 46 samples, total IgG to 34 biotinylated proteins and Cd4-tag alone bound to streptavidin-coated plates was measured using ELISA, as described [22].

12 highly-immunoreactive proteins identified in the immunoreactivity screen were subsequently screened using ELISA [22] in 144 individual samples from the same survey to investigate relationships with infection status, clinical symptoms, and socioeconomic indicators. Samples were randomly selected based on age and infection status in a 3x3 factorial design that included 48 children (5–9 years), 48 adolescents (10–19 years), and 48 adults (20–80 years) either without any *Plasmodium* infections, with a current *P*. *vivax* monoinfection detected by PCR, or with a current *P*. *vivax* monoinfection detected by both PCR and light microscopy (LM). A detailed description can be found in S2 Table. Plasma pools from malaria-naïve Australian and highly-immune PNG adult donors were included on each plate as negative and positive controls, respectively. Samples were tested in duplicate on separate plates.

#### Cohort study

Of the 12 highly-immunoreactive proteins, 6 were selected based on their reactivity in Solomon Islanders and previously reported reactivity in Cambodians [22], as well as their expression levels. These 6 proteins were expressed and purified as described above, along with a Cd4-tag control, and used to measure total IgG in samples from a longitudinal cohort of PNG children described in detail in [27]. Briefly, 264 children aged 1–3 years from a rural area near Maprik, East Sepik Province were enrolled in March-September 2006 and followed for up to 16 months. Children were actively checked for morbidity every 2 weeks, and passive case detection was performed over the entire study period. All PCR+ *P*. *vivax* infections were genotyped to determine the incidence of genetically distinct blood-stage infections acquired during follow-up (i.e., the molecular force of blood-stage infections, molFOB) [28, 29]. Only samples from 230 children who completed follow-up were included in the present study. Luminex bead array assays to measure total IgG were performed as described [30] using plasma and secondary antibody donkey F(ab’)_2_ anti-human IgG Fc R-PE (Jackson ImmunoResearch) diluted 1:100 in PBS. Bead array assays included the same set of controls used for ELISAs and a dilution series of the highly-immune PNG positive control pool to standardize plate-to-plate variations. Samples were tested in singlicate.

### Statistical analysis

#### Immunoreactivity screen

For all ELISA data, an OD cut-off of 0.1 was set as a conservative lower limit based on the plate reader’s limit of accurate detection, and samples with OD values <0.1 were set to 0.1. Differences in population mean antibody levels to proteins and Cd4 alone, and by age groups were assessed using Mann-Whitney U test.

#### Comprehensive screen

Duplicate wells were averaged and OD values for Cd4 subtracted to correct for background. OD values were log_10_-transformed and differences in mean antibody levels by exposure variables assessed using 2-tailed unpaired *t*-test or ANOVA. Multivariate ANOVA models were fitted including all variables that were univariately associated with IgG levels, with the best model determined by backward elimination using Wald’s Chi-square tests for individual variables.

Cut-offs for positivity were set at 2 standard deviations above the mean antibody levels to the negative controls. Differences in the breadth of antibody levels by age and infection status were assessed using negative binomial regression. To estimate seroconversion and seroreversion rates, seroprevalence data were stratified in 5- or 10-year age bins and analysed using reverse catalytic modelling as described elsewhere [31, 32], with the model fitted in a Bayesian framework.

#### Cohort study

To correct plate-to-plate variations, the dilutions of the highly-immune PNG positive control pool were fitted as plate-specific standard curves using a 5-parameter logistic regression model [33]. For each plate, Luminex median fluorescence intensity (MFI) values were interpolated into relative antibody units based on the parameters estimated from the plate’s standard curve. Antibody units ranged from 1.95x10^-5^ (i.e., equivalent to 1:51200 dilution of the immune pool) to 0.02 (1:50). To account for the background reactivity to the Cd4-tag, antibody levels were re-scaled by using linear regression to estimate the antibody levels that would be detected if reactivity to the Cd4-tag was zero, as follows:

log(AB_meas)=log(AB_true)+β*log(Cd4)

where AB_meas = measured antibody level, AB_true = true antibody level to a given antigen, and Cd4 = measured antibody level to the Cd4-tag.

Associations between antibodies and age and exposure were assessed using Spearman rank correlation, and differences with infection using 2-tailed unpaired *t*-test on log_10_-transformed values. Negative binomial GEE models with exchangeable correlation structure and semi-robust variance estimator [34] were used to analyse the relationship between IgG levels and prospective risk of *P*. *vivax* episodes (defined as axillary temperature ≥37.5°C or history of fever in the preceding 48 hours with a current *P*. *vivax* parasitemia >500 parasites/μL). For this, IgG levels were classified into terciles and analyses done by comparing children with low versus medium and high antibody levels. Children were considered at risk from the first day after the initial blood sample was taken. The molFOB, representing individual differences in exposure, was calculated as the number of new *P*. *vivax* clones acquired per year at risk, and square-root transformed for better fit [29]. All GEE models were adjusted for seasonal trends, village of residency, age, and molFOB. In multivariate models that included all antigens that were univariately associated with protection, the best model was determined by backward elimination using Wald’s Chi-square tests for individual variables. To investigate the effect of increasing cumulative IgG levels to the combination of antigens on the risk of *P*. *vivax* episodes, we assigned a score of 0, 1, and 2 to low, medium, and high antibody levels, respectively, and then added up the scores to the 6 antigens to generate a breadth score per child. The breadth score was then fitted as a continuous covariate in the GEE model described above. All analyses were performed using STATA version 12 (StataCorp) or R version 3.2.1 (htpp://cran.r-project.org).

### Ethical statement

Ethical clearance was obtained from the PNG Medical Research and Advisory Committee of the Ministry of Health, Solomon Islands National Health Research Ethics Committee, and the Walter and Eliza Hall Institute. Informed consent was obtained from all participants and in cases of children from their parents or guardians. As approved by the Australian and Solomon Islands’ ethics committees, only verbal consent, documented on each participant’s case report form, was obtained from participants in the cross-sectional survey in Ngella, Solomon Islands. Written informed consent was obtained from the parents or guardians of all children participating in the PNG cohort study.

## Results

### *P*. *vivax* merozoite proteins are targets of natural humoral immunity

**Fig 1:**
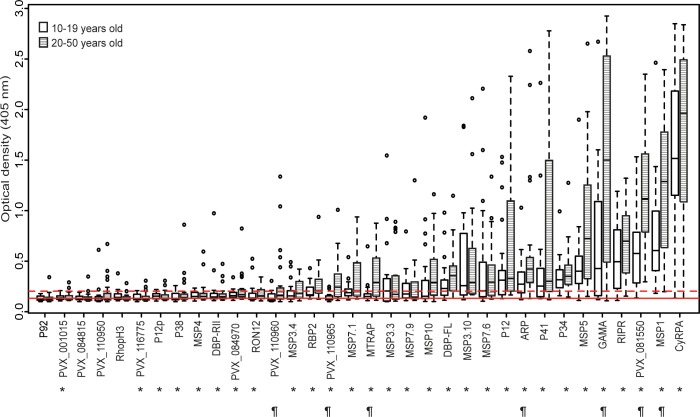
IgG reactivity to 34 *P*. *vivax* merozoite proteins in Solomon Islanders. Boxplots show median optical density (horizontal bar), interquartile range (boxes), range (whiskers), and outliers (open circles). Antibodies to the Cd4-tag are represented as mean (solid red line) and mean + 2 standard deviations (dashed red line). Clear boxes represent adolescents (10–19 years; n = 22;) and striped boxes represent adults (20–50 years; n = 24). Asterisks indicate 29/34 proteins for which the population mean antibody level was significantly higher than to Cd4 alone. ¶ symbols indicate 7/34 proteins for which IgG levels differed significantly between adolescents and adults. *P* values are from Mann-Whitney U tests and were deemed significant if <0.05.

We first investigated whether IgG from 46 *P*. *vivax*-exposed SI individuals recognized antigens from our library. There was a high degree of variability in IgG levels to the different proteins. The population mean antibody level to 85.3% (29/34) of proteins was significantly higher than to Cd4 alone (*P*<0.001–0.018) and these were therefore considered immunogenic (Fig 1). IgG levels were similar between adolescents and adults, except for 7/34 proteins (*P*<0.001–0.032) (Fig 1). We then selected 12 of the most immunogenic proteins, MSP3.3, MSP10, MSP7.6, MSP3.10, P12, ARP, P41, MSP5, GAMA, RIPR, MSP1, and CyRPA, for further analysis.

### The breadth of antibodies increases with age and infection

**Fig 2:**
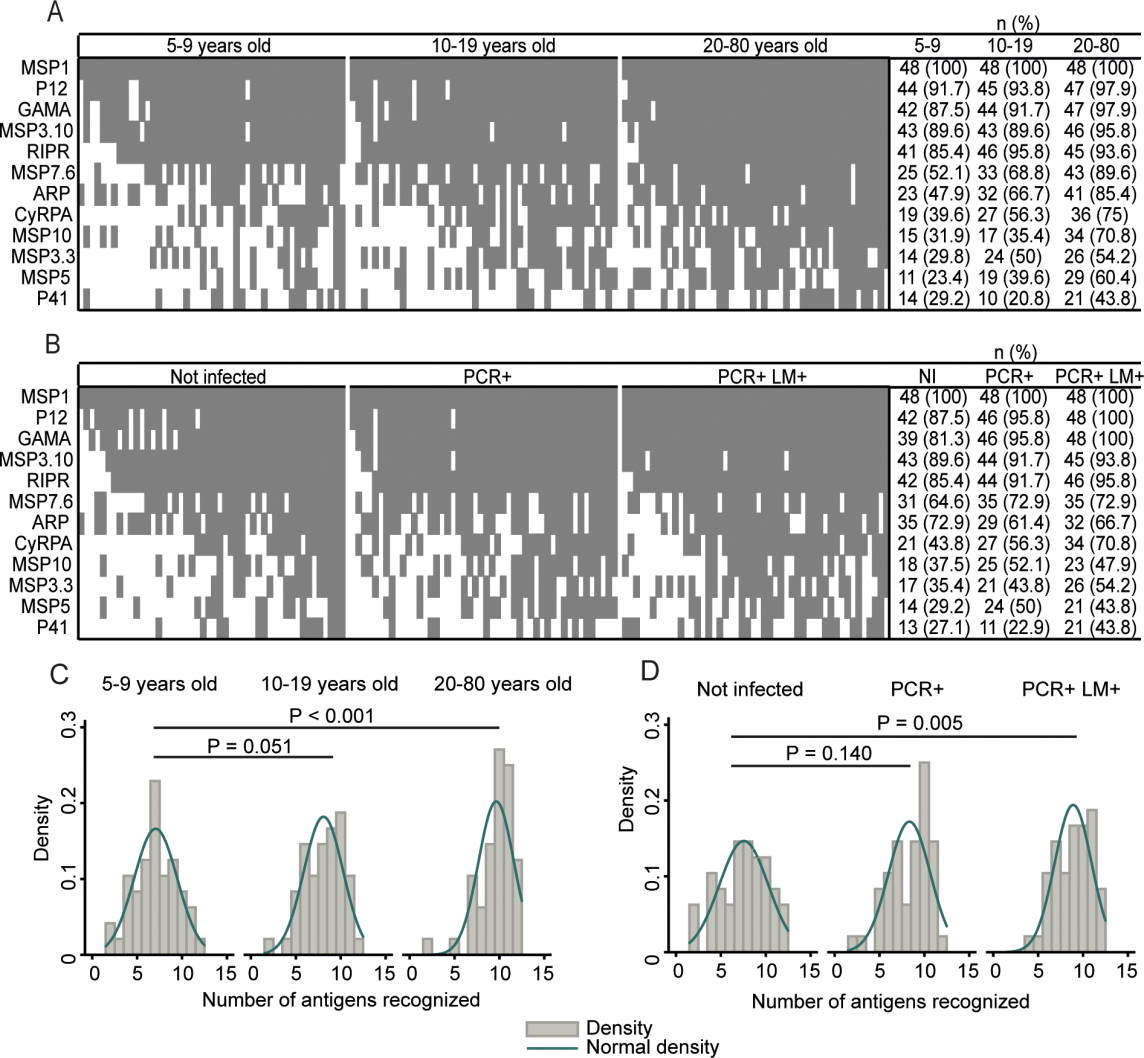
Seroprevalence profiles for 12 *P*. *vivax* merozoite antigens in Solomon Islanders. For each antigen, positivity was defined as 2 standard deviations above the mean optical density from Australian malaria-naïve adults. (A) and (B) show heatmaps of seroprevalence by age group (5–9 years, n = 48; 10–19 years, n = 48; 20–80 years, n = 48) and by *P*. *vivax* infection status (Not infected, n = 48; PCR+, n = 48; PCR+ LM+ n = 48), respectively. Each row shows antibodies observed in all individuals to a single protein, and each column shows antibodies observed in a single individual to all proteins. (C) and (D) show density plots representing the number of antigens recognized in each of the age and infection status groups, respectively. Normally-distributed density curves are shown in dark green. *P* values are from negative binomial regression and were deemed significant if <0.05.

We tested these 12 proteins against a larger panel of 144 SI samples. Antibody seroprevalence ranged from 31.3% (P41) to 100% (MSP1) (S1 Table). MSP1 was recognized by 100% of samples, and P12, GAMA, MSP3.10, and RIPR were each recognized by at least 85% of the children, 89% of the adolescents, and 93% of the adults (Fig 2A). Children recognized fewer proteins (mean 7.06) than adults (mean 9.65; *P*<0.001) or adolescents (mean 8.08; *P* = 0.051) (Fig 2C). Similarly, individuals with a current infection detected by both PCR and LM (mean 8.90; *P* = 0.005), but not those with only PCR+ infections (mean 8.33; *P* = 0.140), had antibodies to significantly more proteins than noninfected individuals (mean 7.56), suggesting a limited effect of recent infections even in adults (Fig 2B and 2D). We applied a serocatalytic model to the seroprevalence data to investigate the kinetic (seroconversion and seroreversion rates) of IgG to antigens with <85% prevalence in children. The estimates are shown in S1 Text.

### Antibody levels reflect both cumulative and current exposure

**Fig 3:**
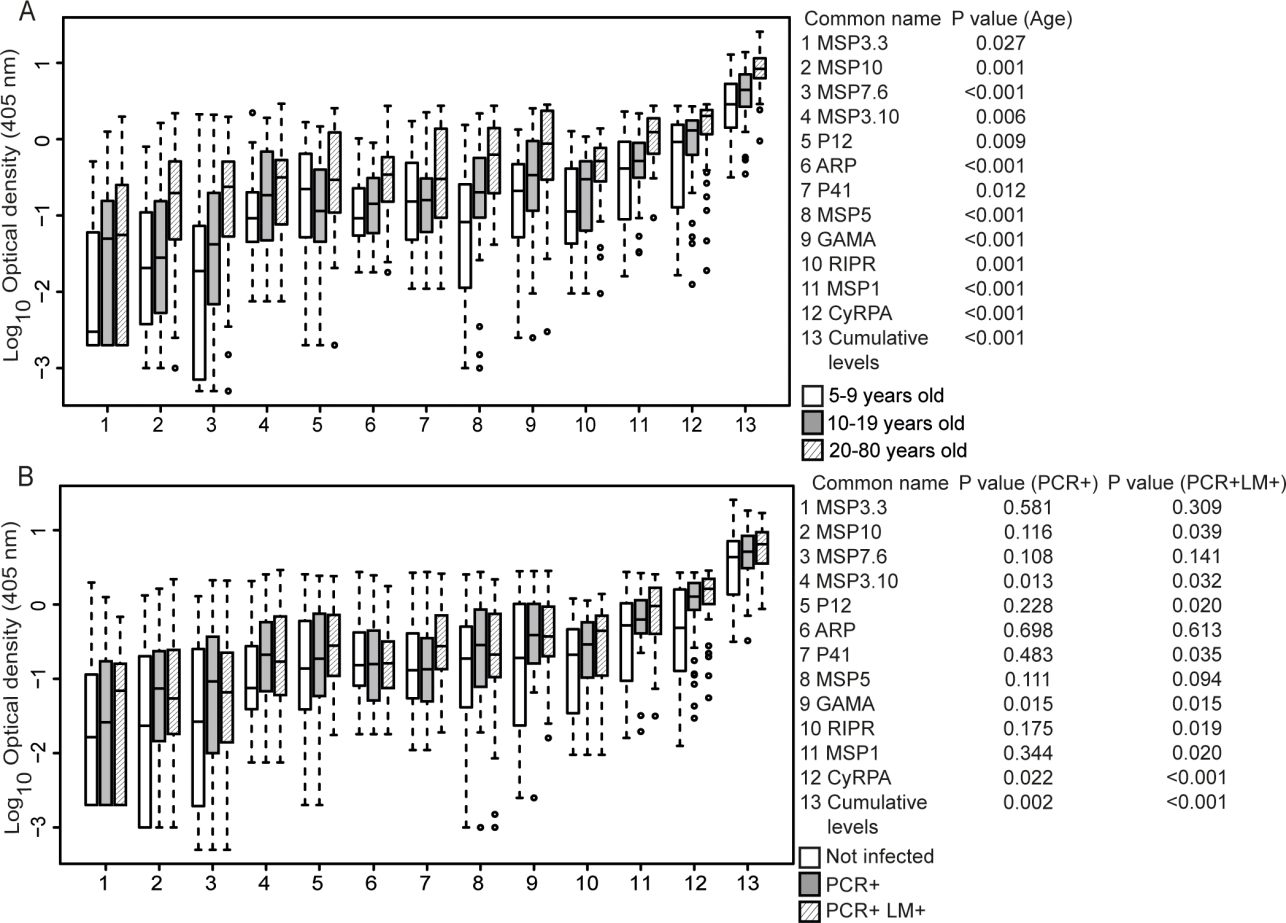
Magnitude of IgG levels to 12 *P*. *vivax* merozoite proteins in Solomon Islanders. Boxplots show median optical density (horizontal bar), interquartile range (boxes), range (whiskers), and outliers (open circles). (A) shows data according to age group (5–9 years, n = 48, clear boxes; 10–19 years, n = 48, grey boxes; 20–80 years, n = 48, striped boxes). (B) shows data according to *P*. *vivax* infection status (Not infected, n = 48, clear boxes; PCR+, n = 48, grey boxes; PCR+ LM+, n = 48, striped boxes). *P* values are from ANOVA and were deemed significant if <0.05.

The magnitude of the cumulative levels (i.e., sum of IgG levels to all antigens, per individual), as well as IgG levels to all individual antigens were strongly associated with age, increasing significantly from children to adults (*P*<0.001–0.027) (Fig 3A). The magnitude of the cumulative antibody levels was also increased in the presence of current infection at either lower (PCR+ only, *P* = 0.008) or higher parasite density (PCR+ LM+, *P* = 0.001) (Fig 3B). Individually, lower-density infections were associated with higher IgG levels to CyRPA (*P* = 0.022), GAMA (*P* = 0.015), and MSP3.10 (*P* = 0.013) only, while higher-density infections were associated with higher IgG levels to a larger number of antigens: CyRPA (*P*<0.001), GAMA (*P* = 0.015), MSP3.10 (*P* = 0.032), P12 (*P* = 0.020), P41 (*P* = 0.035), MSP1 (*P* = 0.020), MSP10 (*P* = 0.039), and RIPR (p = 0.019) (Fig 3B). These data indicate that the antigens in our panel are good markers of cumulative exposure, and that some of them are also markers of current infection.

### Predictors of antibody levels to *P*. *vivax* merozoite proteins

In multivariate analysis, age remained strongly associated with increased cumulative and IgG levels to all individual antigens (*P*<0.001–0.023) (S2 Table). Lower-density infections remained associated with increased IgG levels to CyRPA (*P* = 0.012) and GAMA (*P* = 0.007), and higher-density infections remained associated with increased IgG levels to CyRPA (*P*<0.001), GAMA (*P* = 0.005), P12 (*P* = 0.017), P41 (*P* = 0.031), MSP1 (*P* = 0.014), and RIPR (*P* = 0.012). For MSP5, IgG levels were higher only in adults with a current infection (*P* = 0.022) (S2 Table).

We compared IgG levels against a large number of other epidemiological variables (e.g., region, clinical symptoms, and socioeconomic indicators), but none of them were significantly associated with differences in antibody levels for any antigen (S2 Table). The use of ITNs was the only variable that had any significant association, with the use of ITNs in previous years associated with reduced IgG levels to RIPR (*P* = 0.030), MSP1 (*P* = 0.024), and MSP3.3 (*P* = 0.015) (S2 Table). If ITN use is considered a marker for exposure, this also indicates that the levels of antibodies targeting these antigens are particularly sensitive to recent exposure.

### Associations of antibody levels with cumulative and current exposure in PNG children

To establish whether these associations were population-specific or more broadly generalizable, we tested IgG levels to a subset of 6 antigens, chosen on the basis of immunoreactivity and expression levels, in a sub-cohort of 230 PNG children. The median age of the population was 1.7 years (IQR 1.3–2.5), and the prevalence of *P*. *vivax* infection at baseline was 55% by PCR. IgG levels to ARP, CyRPA, and PVX_081550 were positively associated with age (r = 0.15–0.25; *P* = 0.001–0.027). For PVX_081550, stronger increases in IgG with age were observed in children without current infections (r = 0.33; *P*<0.001) than with current infections (r = 0.18; *P* = 0.048) (S3 Table). A current *P*. *vivax* infection was associated with higher IgG levels to CyRPA (*P*<0.001), P12 (*P*<0.001), P41 (*P* = 0.001), and PVX_081550 (*P* = 0.001) (S3 Table). When considering cumulative exposure as a product of age and the number of *P*. *vivax* infections acquired over time (molFOB), increasing IgG levels with cumulative exposure to PVX_081550 (r = 0.41 *P*<0.001) and CyRPA (r = 0.14, *P* = 0.032) are observed in children without current infections (S3 Table).

### Antibody levels and risk of *P*. *vivax* malaria

**Fig 4:**
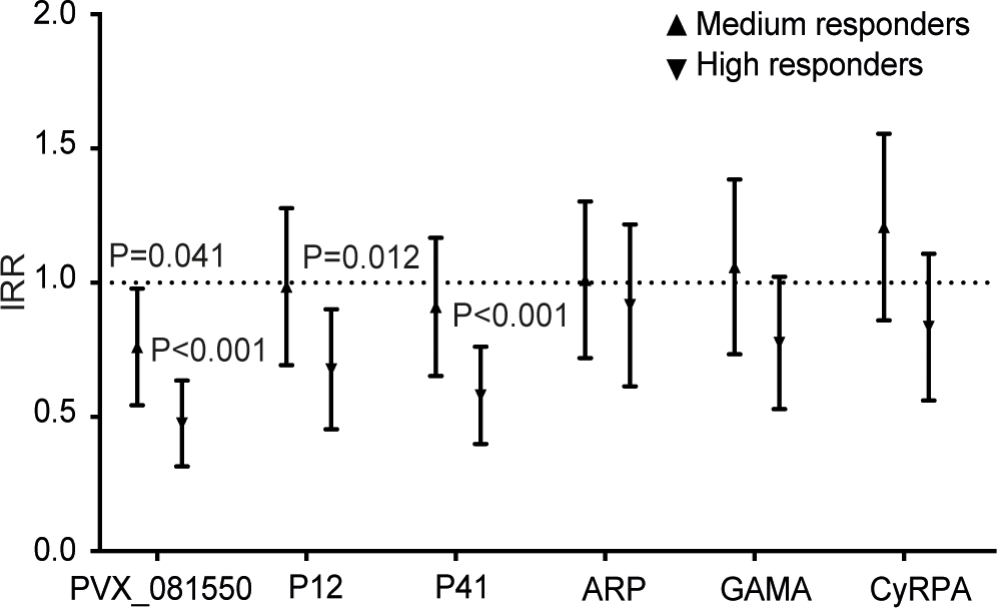
IgG to 6 *P*. *vivax* merozoite proteins and risk of clinical malaria in PNG children. Data are plotted as incidence rate ratios and 95% confidence intervals, adjusted for exposure (molFOB), age, season, and village of residency. Clinical malaria was defined as axillary temperature ≥37.5°C or history of fever in the preceding 48 hours with a current *P*. *vivax* parasitemia >500 parasites/μL. *P* values are from negative binomial GEE models and were deemed significant if <0.05.

During the 16 months of follow-up of the PNG cohort, children experienced an IRR of 1.25 (_95%_CI 1.08–1.45) malaria episodes with *P*. *vivax* >500 parasites/μL/year at risk. We applied the unadjusted GEE model to test whether responses to specific antigens were associated with a reduced risk of infection. Children with high levels of IgG to PVX_081550 (IRR_H_ 0.41; *P*<0.001) and P41 (IRR_H_ 0.63; *P* = 0.019) both had a significantly lower risk of clinical *P*. *vivax* malaria (Table 1). When adjusting for confounders, medium and high levels of IgG to PVX_081550 (IRR_M_ 0.74, *P* = 0.041; IRR_H_ 0.46, *P*<0.001), and high IgG levels to P41 (IRR_H_ 0.56; *P*<0.001) and P12 (IRR_H_ 0.65; *P* = 0.012) were associated with protection. No association with protection was observed for levels of IgG to GAMA, CyRPA, and ARP (Fig 4; Table 1).

**Table 1:** Association between levels of IgG to *P*. *vivax* merozoite proteins and protection against clinical malaria (with parasite density >500/μL of blood) in Papua New Guinean children.

Antigen	uIRR	95%CI		P value	aIRR*	95%CI		P value
PVX_081550 M	0.76	0.54	1.05	0.10	0.74	0.55	0.99	0.041
PVX_081550 H	0.41	0.29	0.60	<0.001	0.46	0.33	0.64	<0.001
ARP M	0.93	0.66	1.32	0.68	0.98	0.73	1.32	0.91
ARP H	1.00	0.69	1.46	0.98	0.88	0.63	1.23	0.47
GAMA M	1.12	0.80	1.57	0.51	1.03	0.75	1.40	0.87
GAMA H	0.82	0.55	1.23	0.34	0.75	0.54	1.04	0.08
P41 M	0.96	0.68	1.36	0.83	0.89	0.67	1.18	0.41
P41 H	0.63	0.43	0.93	0.019	0.56	0.41	0.77	<0.001
P12 M	1.05	0.75	1.47	0.79	0.96	0.71	1.29	0.77
P12 H	0.69	0.47	1.02	0.06	0.65	0.47	0.91	0.012
CyRPA M	1.06	0.76	1.46	0.74	1.17	0.88	1.57	0.28
CyRPA H	0.88	0.59	1.30	0.52	0.81	0.58	1.12	0.20

Abbreviations: M = Medium antibody levels; H = High antibody levels; 95%CI = 95% confidence interval; uIRR = Unadjusted incidence rate ratio; aIRR = Adjusted incidence rate ratio.

*Adjusted for individual differences in exposure (molFOB), age, village of residence, and season.

*P* values, uIRR, and aIRR from negative binomial GEE models. *P* values <0.05 were deemed significant.

IgG levels to the 3 antigens associated with protection were significantly correlated (r = 0.34–0.66; *P*<0.001) (S4 Table), suggesting co-acquisition. In multivariate analyses, only high levels of IgG to PVX_081550 remained strongly associated with reduced risk of *P*. *vivax* episodes (IRR_H_ 0.54; *P* = 0.001), indicating that this antigen may be a key target of natural immunity or a good marker of immunity. There were no significant associations between levels of IgG to any of these 3 antigens and risk of clinical episodes caused by *P*. *falciparum* with any parasite density (IRR 0.92–1.18; *P*>0.10) (S6 Table).

### Protection increases with increasing antibody repertoire

There was a very strong association between increasing antibody repertoire and increase in protection. Each increase in 1 unit of the breadth score (described in Methods) was associated with a reduction of approximately 7% in the risk of *P*. *vivax* episodes (IRR 0.93; 95%CI 0.90–0.97; *P* = 0.001). However, once we accounted for differences in IgG to PVX_081550, the breadth effect was no longer significant (IRR 0.98; 95%CI 0.93–1.04; *P* = 0.49), while high levels of IgG to PVX_081550 remained associated with protection (IRR_H_ 0.51; 95%CI 0.33–0.80; *P* = 0.004). This finding suggests that IgG level to PVX_081550 is a key marker of protective immunity.

## Discussion

The discovery and rational prioritization of *P*. *vivax* proteins as candidates for inclusion in a future *P*. *vivax* vaccine would be greatly facilitated by a comprehensive and systematic characterization of antibody response to *P*. *vivax* antigens in exposed individuals. Although epidemiological associations do not necessarily denote causality, the identification of such ‘protective’ antibody targets in naturally exposed individuals can be used to prioritize antigens or antigen combinations before testing their efficacy and thus potential vaccine suitability in functional studies. Such sero-epidemiological discovery and down-selection are particularly important for *P*. *vivax*, where the lack of stable *in-vitro* culture and genetic manipulation techniques [6] make functional studies and biology-drive discovery difficult, low throughput, and thus very expensive. To date, only a very small number of *P*. *vivax* antigens, such as DBP, MSP1, MSP3, MSP9, and AMA1 have been investigated [21, 35, 36]. The complexity of naturally-acquired immunity against *P*. *vivax* [37] and the likelihood that it’s multifactorial and involves antibodies against several antigenic targets, unlikely to be identified in only one study, highlight the importance of conducting more screening studies. Investigating the large number of potential targets found in the parasite proteome, however, has been constrained in large part by the difficulty of producing natively-folded recombinant *P*. *vivax* antigens. We have leveraged our recent development of a large library of immunoreactive merozoite surface and secreted entire ectodomain proteins [22] to perform systematic studies of reactivity to *P*. *vivax* blood-stage antigens in 2 Asia-Pacific populations.

The vast majority of these proteins (28/34) were recognized by plasma IgG from asymptomatic (including noninfected) adolescent and adult Solomon Islanders. Of these, 27 were also recognized by pooled IgG from Cambodian *P*. *vivax* malaria patients [22]. Although there are individual differences between study populations (e.g., PVX_116675 was only recognized in SI, and PVX_110950 and RhopH3 were only recognized in Cambodia), the use of a large protein library for the first time confirms the broad immunogenicity of a large number recombinant proteins, and also that the pool of potential vaccine targets is much deeper than has been studied to date.

For 12 highly-immunogenic proteins (MSP3.3, MSP10, MSP7.6, MSP3.10, P12, ARP, P41, MSP5, GAMA, RIPR, MSP1, and CyRPA), we confirmed that IgG levels increase more strongly with age, and thus cumulative life-time exposure, than with current infection. Lower and asymptomatic parasitemias are prevalent in SI, a sign that despite significant recent reductions in transmission, residents have acquired significant immunity that is characterized by long-lasting, stable antibody levels. In several studies, antibodies to the *P*. *falciparum* homologs of some of the proteins included in our study were shown to be strongly associated with clinical immunity to *P*. *falciparum* [38, 39]. It is therefore likely that the observed high antibody levels to these *P*. *vivax* proteins contribute to the strong levels of clinical immunity in the SI community.

The associations of clinical immunity with antibodies to 3 antigens (P12, P41, and PVX_081550) were confirmed in a cohort of young, semi-immune PNG children. The observed reductions in risk of *P*. *vivax* malaria were comparable to those associated with high antibody titers to *P*. *vivax* MSP3α and MSP9 [34]. In *P*. *falciparum*, P12 is a GPI-anchored rhoptry protein [40], while P41 is localized to the merozoite surface [41]; together, they form a heterodimer and are thought to be involved in reticulocyte invasion, although neither is essential for parasite growth *in vitro* [42]. Both are strongly recognized by natural immunity, and antibodies have also been associated with clinical protection [38, 39]. It is likely that *P*. *vivax* P12 and P41, which also form a heterodimer [22], have comparable functions.

The protein with the strongest association with protection was the hypothetical protein, PVX_081550. Its *P*. *falciparum* homologue has recently been identified as StAR-related lipid transfer protein [43], able to transfer different lipids between phospholipid vesicles. In *P*. *falciparum*, this protein localizes to the parasitophorous vacuole (PV); there is some evidence that it may be transferred into the apical organelles of mature merozoites, where it may play a role in forming the PV during the invasion process [43]. Although the *P*. *falciparum* protein was also found to be immunogenic [44], it is unclear whether antibodies to it interfere with parasite function (e.g., block erythrocyte invasion) or are simply elicited by proteins released from the PV upon schizont rupture and thus serve only as markers of an individual’s immune status. Both proteins are polymorphic, with nonsynonymous/synonymous SNP ratios of 1.9–2.3 (PlasmodDBv26 [45]). Further studies are now needed to elucidate the function of both *P*. *falciparum* and *P*. *vivax* StAR-related lipid transfer proteins, and importantly to determine whether antibodies to *P*. *vivax* PVX_081550 are functionally protective or simply a useful marker of a child's overall immune status.

Our studies have confirmed that a large array of *P*. *vivax* merozoite antigens are targets of natural humoral immunity, and that antibodies to little-studied proteins may have equivalent or even stronger associations with reduced malaria risk in naturally exposed populations in comparison to current leading vaccine candidates. Further studies, including both in-depth evaluations of their association with protection in longitudinal cohort studies in other transmission settings and functional studies (to the extent this is currently possible for *P*. *vivax*), will be required to determine the potential of these proteins as vaccine candidates, markers of immune status, markers of cumulative exposure, or some combination thereof.

## Supporting Information

S1 Text Antibody kinetics. (DOCX)

S1 Fig Comparison between antibody seroprevalence data and fits of the serocatalytic model. Data are shown as point estimates (square) with 95% credible intervals (vertical bars) of seroprevalence in 5- or 10-year age bins. The model fit corresponding to the posterior median parameter estimates is shown with the solid line.
(DOCX)

S1 Table Recombinant *P*. *vivax* proteins used in the study. (XLSX)

S2 Table Associations between levels of IgG to 12 *P*. *vivax* merozoite proteins measured by ELISA and infection status, clinical symptoms, and socioeconomic indicators in Solomon Islanders. (XLSX)

S3 Table Associations between levels of IgG to 6 *P*. *vivax* merozoite proteins and age, exposure status, and infection status in Papua New Guinean children. (XLSX)

S4 Table Correlation between IgG levels to 3 *P*. *vivax* merozoite antigens in Papua New Guinean children. (XLSX)

S5 Table Parameter estimates for serocatalytic models. Estimates are provided as median and 95% credible intervals of the posterior distribution. All parameters had uniform prior distributions: λ ~ U(0,10), *ρ* ~ U(0,1).
(XLSX)

S6 Table Association between levels of IgG to *P*. *vivax* merozoite proteins and protection against clinical *P*. *falciparum* malaria episodes (all parasite densities) in Papua New Guinean children. (XLSX)
