# Correction: An antibody screen of a *Plasmodium vivax* antigen library identifies novel merozoite proteins associated with clinical protection

**DOI:** 10.1371/journal.pntd.0013122

**Published:** 2025-05-22

**Authors:** Camila T. França, Jessica B. Hostetler, Sumana Sharma, Michael T. White, Enmoore Lin, Benson Kiniboro, Andreea Waltmann, Andrew W. Darcy, Connie S. N. Li Wai Suen, Peter Siba, Christopher L. King, Julian C. Rayner, Rick M. Fairhurst, Ivo Mueller

The hyperlink in the Data Availability statement is incorrect. The correct hyperlink is: https://datadryad.org/dataset/doi:10.5061/dryad.pn8mn.
