# Supplementary material for: An Antibody Screen of a Plasmodium vivax Antigen Library Identifies Novel Merozoite Proteins Associated with Clinical Protection
Source: PLoS Negl Trop Dis. 2016 May 16;10(5):e0004639. doi: 10.1371/journal.pntd.0004639 (PMC4868274; doi:10.1371/journal.pntd.0004639)
Supplement: S1 Text — (DOCX) [file pntd.0004639.s001.docx]

**Antibody kinetics**

Seroprevalence curves reached a plateau around 20 years of age for most antigens with <85% prevalence in children. Catalytic models estimate seroconversion rates of once every ~10 years for ARP, CyRPA, MSP7.6, and MSP3.10; <1 year for MSP10 and MSP5; and >60 years for P41, reflecting the known high past malaria transmission and exposure in the SI, as well as differences in antigenicity (S1 Fig; Table S5). It was estimated that 50% of the population would serorevert after a period of <1 year for P41; 11-16 years for MSP5 and CyRPA, and 21-57 years for MSP10, ARP, and MSP7.6, suggesting that antibody responses to the majority of the antigens have long half-life in plasma (Table S5).
