# Supplementary material for: An Antibody Screen of a Plasmodium vivax Antigen Library Identifies Novel Merozoite Proteins Associated with Clinical Protection
Source: PLoS Negl Trop Dis. 2016 May 16;10(5):e0004639. doi: 10.1371/journal.pntd.0004639 (PMC4868274; doi:10.1371/journal.pntd.0004639)

Figure S1. Comparison between data on antibody seropositivity and fits of the serocatalytic model.


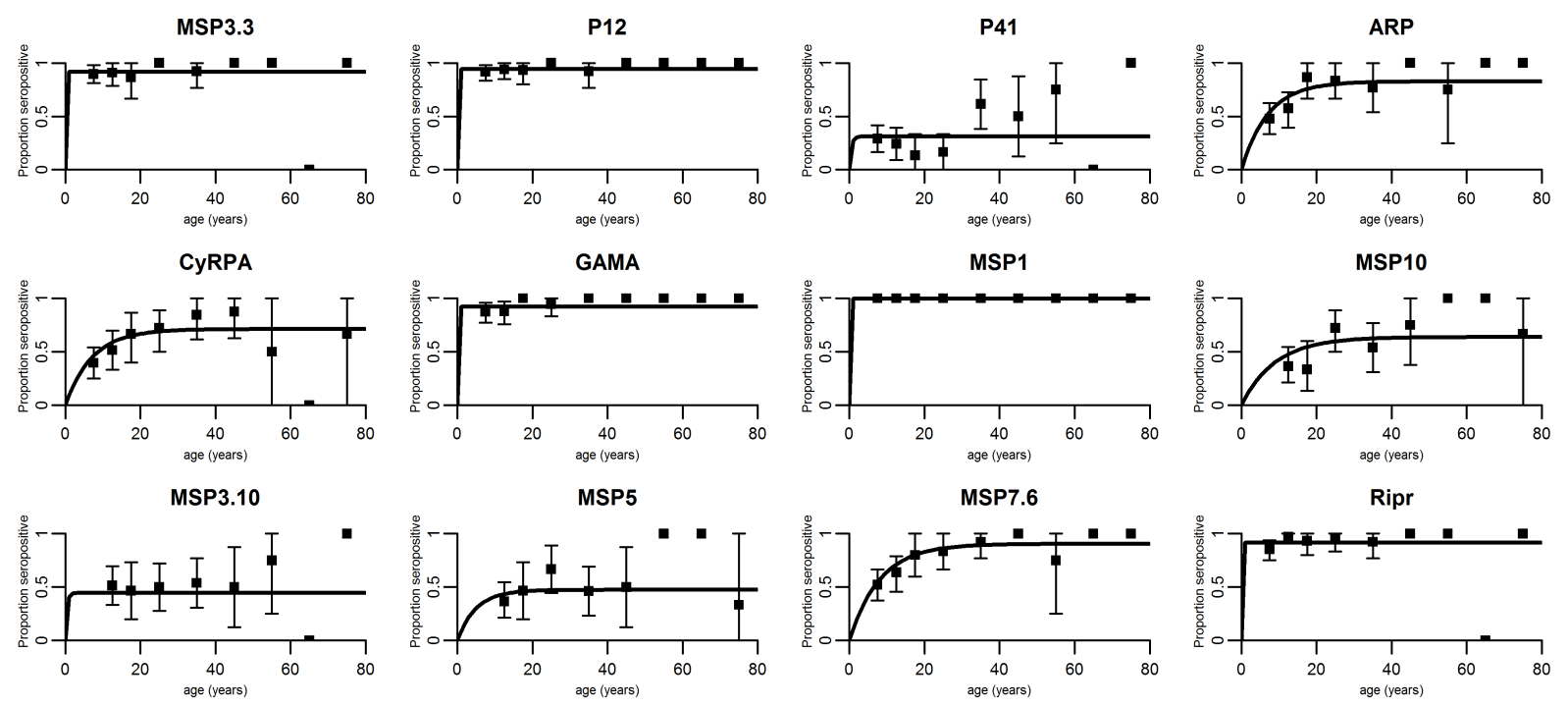

Supplement: S1 Fig — Data are shown as point estimates (square) with 95% credible intervals (vertical bars) of seroprevalence in 5- or 10-year age bins. The model fit corresponding to the posterior median parameter estimates is shown with the solid line. (DOCX) [file pntd.0004639.s002.docx]
